# Cellular reprogramming through mitogen-activated protein kinases

**DOI:** 10.3389/fpls.2015.00940

**Published:** 2015-10-29

**Authors:** Justin Lee, Lennart Eschen-Lippold, Ines Lassowskat, Christoph Böttcher, Dierk Scheel

**Affiliations:** ^1^Department of Stress and Developmental Biology, Leibniz Institute of Plant BiochemistryHalle/Saale, Germany; ^2^Federal Research Centre for Cultivated Plants, Ecological Chemistry, Julius Kühn Institute, Plant Analysis and Stored Product ProtectionBerlin, Germany

**Keywords:** MAPK substrates, phosphorylation, phosphoproteome, metabolome, chemical defense

## Abstract

Mitogen-activated protein kinase (MAPK) cascades are conserved eukaryote signaling modules where MAPKs, as the final kinases in the cascade, phosphorylate protein substrates to regulate cellular processes. While some progress in the identification of MAPK substrates has been made in plants, the knowledge on the spectrum of substrates and their mechanistic action is still fragmentary. In this focused review, we discuss the biological implications of the data in our original paper (Sustained mitogen-activated protein kinase activation reprograms defense metabolism and phosphoprotein profile in *Arabidopsis thaliana;* Frontiers in Plant Science 5: 554) in the context of related research. In our work, we mimicked *in vivo* activation of two stress-activated MAPKs, MPK3 and MPK6, through transgenic manipulation of *Arabidopsis thaliana* and used phosphoproteomics analysis to identify potential novel MAPK substrates. Here, we plotted the identified putative MAPK substrates (and downstream phosphoproteins) as a global protein clustering network. Based on a highly stringent selection confidence level, the core networks highlighted a MAPK-induced cellular reprogramming at multiple levels of gene and protein expression—including transcriptional, post-transcriptional, translational, post-translational (such as protein modification, folding, and degradation) steps, and also protein re-compartmentalization. Additionally, the increase in putative substrates/phosphoproteins of energy metabolism and various secondary metabolite biosynthesis pathways coincides with the observed accumulation of defense antimicrobial substances as detected by metabolome analysis. Furthermore, detection of protein networks in phospholipid or redox elements suggests activation of downstream signaling events. Taken in context with other studies, MAPKs are key regulators that reprogram cellular events to orchestrate defense signaling in eukaryotes.

## Introduction

Since, plants are part of the ecological basis for oxygen production and food source of most lifeforms on earth, crop yield loss through stress conditions is an increasing threat to food security in view of the ever increasing human population and climate change. As sessile organisms, plants adopt mostly non-motile mechanisms to survive unfavorable conditions such as abiotic stresses or biotic interactions with pests. Understanding how plants sense stress stimuli and transduce these via cellular signaling events to coordinate an appropriate response is a major challenge in current plant research for developing strategies to mitigate agricultural yield loss from (a)biotic stresses.

**Figure 1:**
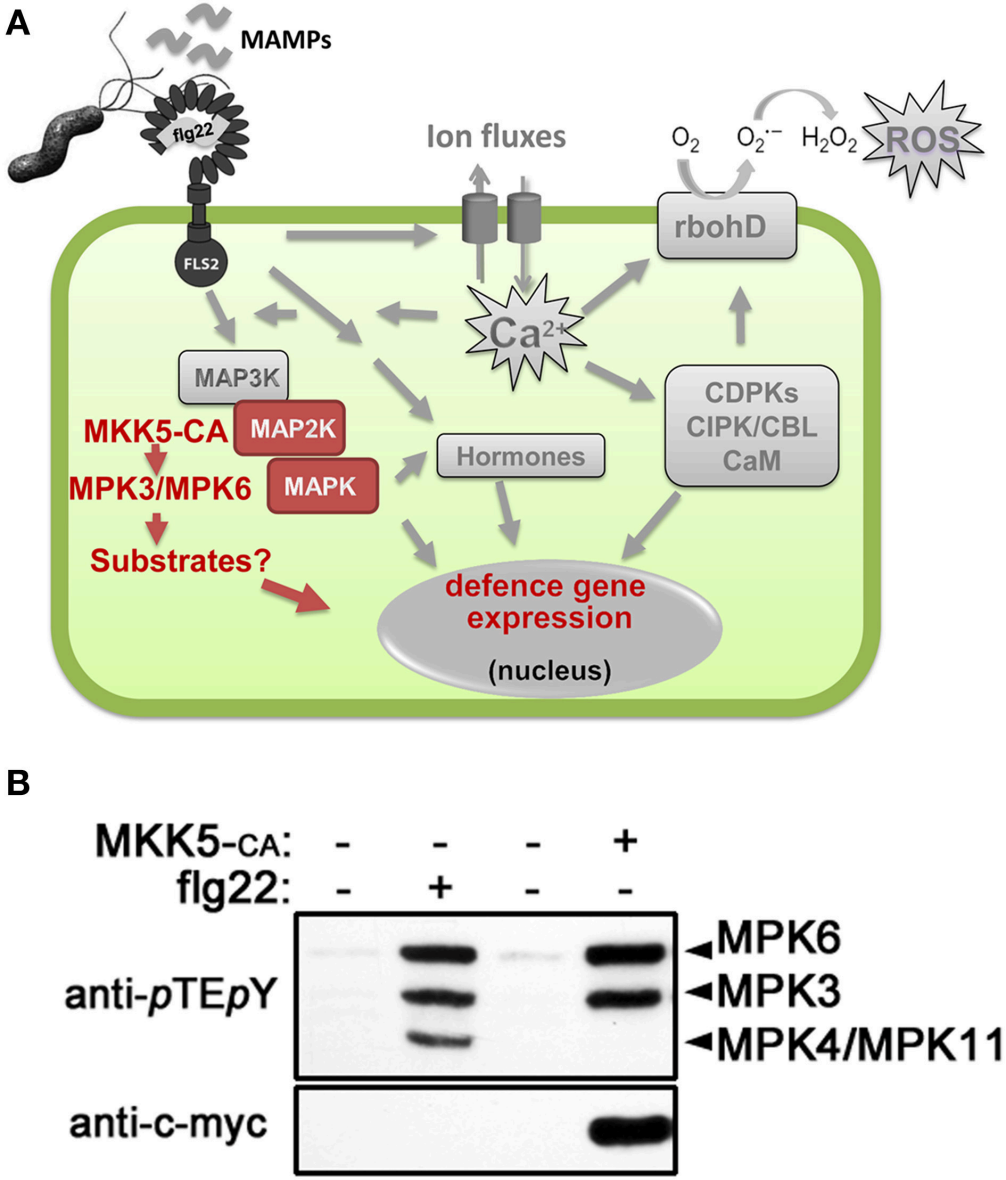
**MAMP (microbe-associated molecular pattern)-induced cellular signaling. (A)** Schematic representation of the MAMP-induced cellular signaling pathway in plant cells, using flg22 peptide as an example of a classical MAMP. See introduction text for description. Highlighted in red are the events generated by *in vivo* activation of the mitogen-activated protein kinases (MAPKs), MPK3 and MPK6, by transgenic expression of a constitutively active MAPK kinase (MKK5-CA). **(B)** Western blot analysis for detecting activated forms of MAPKs in Arabidopsis protoplasts after flg22 treatment or transfection of c-myc-tagged MKK5-CA construct. The identities of the MAPK bands are indicated on the right. Protoplast transfection and western blotting to identify specific activated MAPKs was performed as described (Ranf et al., 2011; Eschen-Lippold et al., 2012; Lassowskat et al., 2014). Abbreviations used: FLS2, flg22 receptor; rbohD, NADPH oxidase responsible for flg22-induced reactive oxygen species (ROS) production; CDPKs, calcium-dependent protein kinases; CBL, calcineurin-B-like protein; CIPK, CBL-interacting protein kinase; CaM, calmodulin.

The sensing of potential pathogens or molecules released by microbes leads to complex signaling series of events, including ion fluxes, oxidative burst, activation of mitogen-activated protein kinase (MAPK) cascades, calcium decoding mechanisms (e.g., Calmodulin, calcium dependent protein kinases, Calcineurin B-like proteins, and their interacting kinases) (Romeis, 2001), hormonal control (Bari and Jones, 2009; Knogge et al., 2009) and defense-related gene expression (Boller and Felix, 2009; Figure 1A). For this review, we will focus on MAPK cascades, which comprise three consecutive kinases—a MAPK kinase kinase (MAPKKK), a MAPK kinase (MKK), and the MAPK itself (Gustin et al., 1998). They play crucial roles in diverse developmental and stress-related adaptation processes—enabling the organism to transduce external stimuli into cellular responses in eukaryotes (Suarez Rodriguez et al., 2010). After treatment with conserved microbe-derived molecules (so-called microbe-associated molecular patterns, MAMPs), two main branches of MAPK cascades have been described in the model plant, *Arabidopsis thaliana*: one involving MKK4/5-MPK3/6 (Asai et al., 2002) and the other, MEKK1-MKK1/2-MPK4 (Ichimura et al., 2006). Introduction of inactive MAPKs or MKKs showed that MPK3/MPK6 control defense gene expression positively (Asai et al., 2002; Kroj et al., 2003). By contrast, MPK4 negatively regulates defense, since the *mpk4* mutant had enhanced expression for subsets of *pathogenesis-related* genes and is more resistant to biotrophs (Petersen et al., 2000). Hence, MAPK cascades are involved in defense signaling and are critical for coordinating an adequate defense response.

Using activity-based in-gel or immunoblot assays to visualize activated MAPKs, three prominent bands representing MPK3, MPK4, and MPK6 are detected after MAMP treatment (Figure 1B) and hence, most work has emphasized on these three MAPKs. However, other MAPKs appear to be also MAMP-activated. MPK11, with a similar size as MPK4, was shown to be a fourth MAMP-activated MAPK (Bethke et al., 2012; Eschen-Lippold et al., 2012). This was validated in a recent independent study where additionally, MPK1 and MPK13 may also be weakly activated by MAMPs (Nitta et al., 2014).

A current challenge in MAPK research is to identify direct, *in vivo* MAPK substrates, their respective phosphorylation sites and elucidate how phosphorylation controls downstream signaling (Rasmussen et al., 2012). To this end, we performed phosphoproteomics studies on plants with simulated *in vivo* activation of MPK3 and MPK6 (Lassowskat et al., 2014), which was achieved by transgenic expression of a constitutively-active MKK5 (Lee et al., 2004). Unlike global phosphoproteomics (Benschop et al., 2007), this approach should deliver a less complex phosphoproteome as all phosphoproteins induced during the signaling steps between MAMP receptor till the MKK step are excluded (see scheme in Figure 1A). Furthermore, such a strategy leads to only two activated MAPKs (Figure 1B) and enables us to focus on the direct substrates (and other downstream phosphoproteins) of only MPK3/MPK6. We reported a total of 538 putative MPK3/MPK6 substrates (see Table S14 of Lassowskat et al., 2014) that were detected after a phosphoprotein enrichment procedure developed for green tissues (Lassowskat et al., 2013). The necessary steps/strategies to validate true MAPK substrates have been discussed and also performed for a few selected candidates in our original publication. In this focused review, we will assess a more global implication of MAPKs' role in signaling based on these 538 putative MAPK substrates and phosphoproteins.

**Figure 2:**
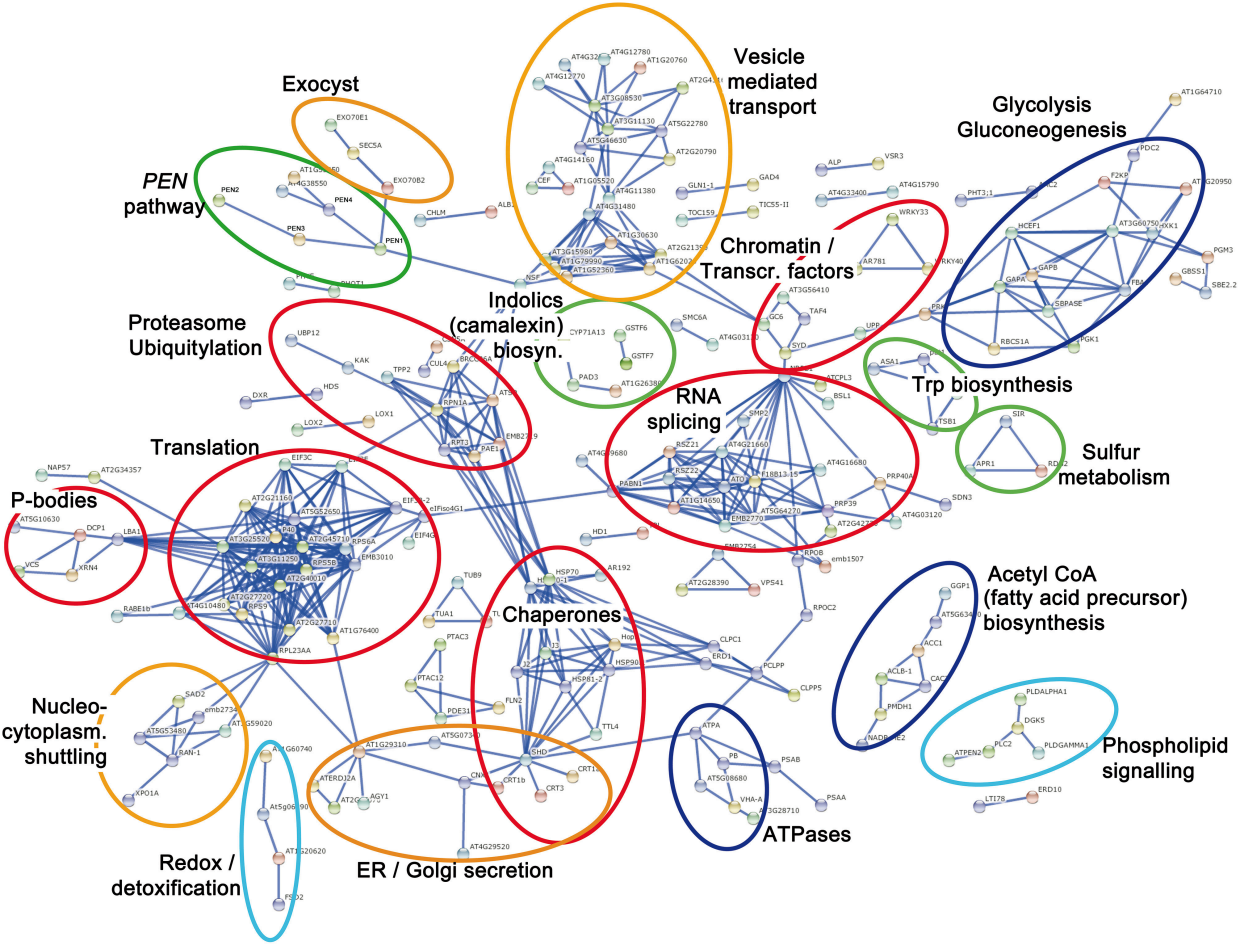
**Protein interaction network based on 538 putative MPK3/MPK6 substrates and downstream phosphoproteins**. The protein network was plotted with STRING 10.0 (high confidence score 0.9, confidence view). Each node represents a protein labeled by its protein name abbreviation or the Arabidopsis locus identifier. Protein nodes without edges are masked and not displayed. The main protein clusters relevant for the four presented Key Concepts are color-coded. Red = (Key Concept 1: Transcriptional, post-transcriptional, translational, and post-translational control); orange = (Key Concept 2: Cellular re-compartmentalization); green (Key Concept 3: Chemical defense); and light blue = (Key Concept 4: Interplay with downstream cellular signaling). Additionally, dark blue = proteins for energy or precursor supply that support processes of one of the other key concepts.

To display the functional protein association network, we used the STRING (version 10) algorithm, which extracts information from curated data (from Biocarta, BioCyc, GO, KEGG, and Reactome databases) and experimental evidences (from BIND, DIP, GRID, HPRD, IntAct, MINT, and PID databases; Jensen et al., 2009; Franceschini et al., 2013). Based on protein-protein interactions, co-expression, or phyletic profiles (i.e., co-occurrence of orthologs in other organisms), a protein network for the 538 proteins was generated. To simplify the visualization, a stringent STRING score of 0.9 (max. = 1.0) was chosen to depict a “high confidence” network and protein nodes without any edges were not displayed. Thus, only the putative MPK3/MPK6 substrates (or downstream phosphoproteins) that can be organized into protein networks are depicted (Figure 2). By categorizing these network clusters, one may infer the coordinated activities of the phosphoproteins/substrates downstream of MPK3/MPK6. In the following sections, we will generalize these into four key concepts to summarize how MAPKs, in general, reprogram cellular biochemical activities to coordinately mount an appropriate (defense) response to stimuli.

## MAPKs control transcription and translation at multiple levels

It is well known that MAPKs target transcription factors to promote gene expression in various eukaryotes including yeast (Bao et al., 2004), animal (Kim-Kaneyama et al., 2000), and plant systems (Meng and Zhang, 2013). Indeed, our and similar studies picked up several WRKY transcription factors (Hoehenwarter et al., 2012; Lassowskat et al., 2014) and associated VQ-motif containing proteins (Pecher et al., 2014). Most transcription factors are not well clustered in protein networks due to limited knowledge of their interactors. Nevertheless, presumably due to their co-expression pattern (Wan et al., 2004), WRKY33 and WRKY40 are organized into a network and can be broadly categorized together with proteins involved in “chromatin remodeling and transcription regulation.” The most conspicuous members in this group are TATA-box associated factor 4 (AT5G43130), the large subunit of RNA polymerase II (RNAP II, AT4G35800) and “C-terminal domain (CTD) phosphatase-like 3” (CPL3, AT2G33540). Recently, it was shown that two antagonistic pathways modulate the activity of RNAP II to control host immune gene expression (Li et al., 2014). MAMP-induced MAPK activates cyclin-dependent kinase C, which then phosphorylates the RNAP II CTD to stimulate transcription. Negative regulation is realized by CPL3 counteracting the RNAP II activation through dephosphorylation of Ser-2 and/or Ser-5 of the heptad repeats YSPTSPS in the CTD of the RNAP II subunit (Li et al., 2014). Another member in this cluster is SPLAYED (AT2G28290), a catalytic component of the chromatin structure remodeling complex. SPLAYED is known to regulate stress and particularly immune responses (Walley et al., 2008; Johnson et al., 2015). While not clustered into the same network by STRING, a SPLAYED-like protein, BRAHMA, which has overlapping and partially redundant functions (Bezhani et al., 2007), was identified in our study, and also in a previous *in vitro* kinase assay as a MAPK substrate (Feilner et al., 2005). Recent chromatin immunoprecipitation studies showed BRAHMA binding directly the chromatin of the regulated genes (Yang et al., 2015). Altogether, this cluster of putative phosphoproteins suggests a phospho-mediated chain of reactions for chromatin-remodeling, nucleosome positioning and transcriptional regulation of defense-related genes after MAPK activation.

Remarkably, closely associated to this chromatin/transcription cluster is a large collection of proteins involved in RNA splicing. Many Ser/Arg-rich (SR) splicing factors and other proteins with roles in RNA metabolism have been previously identified as phosphoproteins in a phosphoproteomics study and the conserved phosphorylation sites identified in the SR splicing factors resemble that typically targeted by MAPKs (de la Fuente van Bentem et al., 2006). Thus, MAPKs appear to control transcription and proper (or alternative) splicing to deliver the mRNA template for protein translation. Incidentally, the “splicing” group is linked (to the left side, Figure 2) through PABP1 (Polyadenylate-Binding Protein 1, AT5G51120) and the RNA-binding EIFiso4G1 (Eukaryotic Translation Initiation Factor isoform 4G1, AT5G57870) to one of the largest cluster in the network. This consists of various ribosomal protein and translation initiation factor (eIF) subunits, which suggests phospho-modification of the translational machinery—presumably in preparation for protein translation of the newly synthesized mRNAs. This is reminiscent of reports in animal systems where MAPK-associated phosphorylation facilitates assembly of the translation preinitiation complex and to correlate with increased cap-dependent translation (Roux et al., 2007). Associated to the “translation” group is a cluster consisting of processing body (P-body) components. P-bodies are sites of RNA processing or storage, and are involved in RNA degradation of specific mRNAs, mRNA temporary storage for subsequent release and translation and also translation arrest (Anderson and Kedersha, 2009; Maldonado-Bonilla, 2014). Several components of P-bodies have recently been found to be targeted by MAPKs (Xu and Chua, 2012; Maldonado-Bonilla et al., 2014; Roux et al., 2015). Our study confirms some of these known substrates and suggests additional P-body elements as possible MAPK substrates. Hence, many of the putative MAPK substrates (and phosphoproteins) are involved in chromatin remodeling, transcription, RNA metabolism, and translation. Taken together, our study shows that MAPKs control multiple layers of transcriptional, post-transcriptional and translational regulation to provide the cell with proteins, which are the “workhorse” of biochemical events (**Key Concept 1)**.

KEY CONCEPT 1. **MAPKs control the expression and levels of proteins - the workhorse of biochemical events in the cell—at multiple levels (Red clusters in Figure 2).**

These include transcriptional (e.g., transcription factors, chromatin remodeling), post-transcriptional (e.g., RNA splicing and RNA metabolism factors), translational (e.g., translation machinery), and post-translational (e.g., chaperones, proteasome, and ubiquitylation factors) regulation.

In order for these proteins to function, they need to be properly folded. In line with this, a cluster of chaperones, including HSP70, HSC70, HSP90, HSP81-2 and the HSP-interacting co-chaperone Hop2 (AT1G62740), was detected. A caveat here is that the artificial MAPK activation system may have overloaded the translational machinery, producing many unfolded proteins (Walter and Ron, 2011) and therefore requiring more chaperones to be produced. However, several HSPs have been shown to regulate, together with RAR1 or SGT1 co-chaperones, the stability of so-called NB-LRR plant immunity receptor proteins (Hubert et al., 2009; Huang et al., 2014). This is in line of MAPK function in defense regulation. Thus, besides assisting folding of their client proteins, these chaperones may act together with protein degradation pathways to modulate plant immunity. Whether direct phosphorylation by MAPKs (or indirectly through other activated kinases) controls their chaperone activities and/or their interaction with proteasome components remains to be seen.

Altogether, it means that MAPKs control several global transcription/translation steps leading to protein synthesis/accumulation—many of these steps are likely to be common for other signaling pathways and are not restricted to stress pathways. The determinants of which proteins to synthesize in order to channel into defense reactions are probably not displayed in our chosen STRINGS depiction if there is insufficient functional network annotation (or they may fail the high confidence cut-off score chosen to draw the protein networks). Hence, the signaling specificity of MAPKs relies on a coordinated action between certain “specificity” factors and the global cellular reprograming steps proposed in **Key Concept 1**.

## MAPKs mediate stability of some proteins and/or removal of unwanted proteins

The above description of MAPK action emphasizes *de novo* protein synthesis to initiate biochemical processes needed to orchestrate an appropriate defense response. However, Concept 1 can be extended to include protein expression via protein stability control through phosphorylation. In fact, phospho-mediated control of protein stability is a recurring theme in many of the MAPK substrates (Meng and Zhang, 2013). It can either lead to increased or decreased stability of the phosphorylated substrates, as was demonstrated for one of the first plant MAPK substrates to be identified, where the unstable 1-aminocyclopropane-1-carboxylic acid synthase 6 (ACS6), a rate-limiting enzyme for ethylene biosynthesis, is stabilized after MAPK phosphorylation (Liu and Zhang, 2004). In contrast, mutations in the phospho-sites of the ethylene response factor, ERF104, render it more unstable after MAMP treatment (Bethke et al., 2009). Similarly, MAMP treatment has been shown to reduce the levels of two other classes of MPK3/MPK6 substrates, tandem zinc finger protein 9 (TZF9) (Maldonado-Bonilla et al., 2014) and members of the MPK3/6-targeted VQ motif-containing proteins (MVQs; Pecher et al., 2014). In the case of the MVQ1 protein, its removal through degradation is proposed to reduce cellular repressor levels and “liberate” its interacting WRKY transcription factors to trigger defense gene expression (Pecher et al., 2014; Weyhe et al., 2014). WRKY33, is one of the MVQ1-interacting WRKYs, and, is itself also phosphorylated by MPK3/MPK6 (Mao et al., 2011; Lassowskat et al., 2014). Besides interacting with multiple MVQs (Pecher et al., 2014), WRKY33 interacts with the MPK4 substrate, MKS1 (Qiu et al., 2008), and other VQ-motif proteins that are apparently not targeted by MAPKs (Lai et al., 2011). This has led to a hypothesis of the existence of a plethora of protein-protein interaction combinatorial possibilities involving WRKYs, MAPKs, and VQ-motif proteins, which regulate defense gene expression *via* MAPK-(in)dependent mechanisms (Weyhe et al., 2014). Similar mechanisms have been demonstrated in which MAPK-mediated degradation of components control signal specificity in yeast where homo- or hetero-dimerization of specific transcription factors determine signaling specificity into either the mating or filamentous growth pathway (Chou et al., 2004).

The destabilization of many MAPK substrates after phosphorylation can thus be seen as the removal of “unwanted” proteins, e.g., repressor proteins, to stimulate defense gene expression. Such a catabolic process may be supported by the many phosphoproteins detected, which can be categorized in the group of “proteasome and ubiquitylation” (Figure 2). These include the E3-ubiquitin ligase KAKTUS (AT4G38600), the ubiquitin-specific protease 12 (AT5G06600) and various 20S/26S proteasome subunits (e.g., proteins encoded by *AT1G29150, AT1G20200, AT1G53850, AT5G58290*, or *AT2G20580*). Notably, *AT2G20580* encodes the 26S proteasome regulatory subunit N1 (RPN1a) that is required for plant immunity to bacterial and fungal pathogens (Yao et al., 2012). It remains to be seen if the putative phosphorylation of these plant proteasome subunits affects their activities or, *vice versa*, phosphorylation of MAPK substrates alters their affinity for the proteasome complex. In this context, a 19S proteasome subunit also acts cooperatively with the animal MAPK pathway to regulate transcription factors that control cell proliferation in several human cancer cell lines, but its mechanism is still unclear (Pakay et al., 2012). Thus, proteasome-mediated degradation seems to be a universal conserved eukaryotic mechanism of MAPKs to control the composition of regulatory protein complexes.

## MAPKs mediate remobilizing of proteins (and other substances) to another cellular compartment

One of the earliest observations on animal MAPK studies has been the re-localization of MAPKs from the cytoplasm to the nuclei upon activation (Brunet et al., 1999; Furuno et al., 2001). Similarly, in several plant system, nuclear import has been observed upon MAMP elicitation or through developmental signals (Ligterink et al., 1997; Coronado et al., 2002; Kroj et al., 2003; Lee et al., 2004). The upstream MKK appears to be excluded from the nucleus while the activated MAPKs dissociate from the MKK and translocate into the nucleus (Lee et al., 2004). Nuclear export signal (NES) within MKKs has been shown to retain the MAPK in the cytoplasm and suggests, besides a role as the upstream kinase, a novel function of MKKs as a cytoplasmic anchoring protein for MAPKs (Fukuda et al., 1997). MAPK nuclear translocation depends on the nuclear import machinery (Adachi et al., 1999) and there is evidence for direct interaction with nuclear pore complex proteins (Matsubayashi et al., 2001) such as importin (Ferrigno et al., 1998). Among the MPK3/MPK6 substrates and downstream phosphoproteins, we identified a cluster of proteins involved in nucleocytoplasmic shuttling (Figure 2, orange circle at the bottom left). This includes RAN1 (AT5G20010), exportin 1a (AT5G17020), importin SAD1 (AT2G31660), a homolog of the human nuclear transport protein KPNB1 (AT5G53480), and importin-α export receptor (AT3G59020). These candidate phosphoproteins are suggestive of a MAPK-induced regulation of the nuclear import-export machinery for subcellular distribution of MAPKs and possibly other proteins (e.g., MAPK substrates, see below). Thus, a second key concept evident from our study is the MAPK control of re-compartmentalization of proteins (and possibly other substances) and it is logical to assume that this remobilization is crucial for action or function of the transported factors **(Key Concept 2)**.

KEY CONCEPT 2. **MAPKs control re-compartmentalization and cellular localization of proteins (or substances) to their site-of-action or -function (Orange clusters in Figure 2).**

Earlier studies suggest the nucleus as a site for signal termination by sequestration away from the MKKs and inactivation of p42/p44 MAP kinases by phosphatases (Volmat et al., 2001). However, there are more examples where nuclear translocation serves to allow MAPKs to target nuclear proteins such as transcription factors or chromatin-associated factors. Several such plant MAPK-targeted nuclear factors have been illustrated above or reviewed recently (Rasmussen et al., 2012; Meng and Zhang, 2013). Alternatively, MAPKs may target cellular proteins to induce nuclear translocation; e.g., the bZIP transcription factor VIP1 is phosphorylated by MPK3, which consequently leads to the relocalization of VIP1 from the cytoplasm to the nucleus where it induces the expression of *pathogenesis-related-1* gene (Djamei et al., 2007). In either scenarios, the movement of either the kinase or its substrate(s) into the nucleus serves to target site- or compartment-specific processes. In the above example, this is to activate gene transcription.

In addition to organelles, a proteomics study has uncovered re-compartmentalization of immunity-related membrane components after MAMP elicitation (Keinath et al., 2010). A prominent example is the flg22 receptor, FLS2 (Robatzek et al., 2006) that detects a 22 amino acid motif of bacterial flagellin (Gómez-Gómez and Boller, 2002). Ligand-induced endocytic trafficking of MAMP receptors controls their degradation or perhaps also the recycling of receptors back to the plasma membrane (Ben Khaled et al., 2015). In support of this, we detected a large cluster of proteins involved in vesicle-mediated transport after mimicking *in vivo* MPK3/MPK6 activation (Figure 2). This includes the vesicle-fusing ATPase NSF1 (AT4G04910) and multiple proteins of the coatomer/clathrin-mediated vesicle transport [e.g., α- (AT2G21390), β- (AT4G31480, AT1G79990), β2- (AT1G52360, AT3G15980) or ε- (AT1G30630) subunits of coatomer protein; α- (AT5G22780, AT1G62020), β- (AT4G11380, AT4G11380), or μ- (AT5G46630, AT2G20790) adaptins; auxilin-like proteins (AT4G12770, AT4G12780); clathrin heavy chain I (AT3G11130); clathrin heavy chain II (AT3G08530); and ENTH/ANTH/VHS superfamily proteins (AT4G32285, AT2G43160)]. In addition, a separate cluster of at least three proteins (Exo70B2, Exo70E1, and Sec5A) are exocyst components. The octameric exocyst protein complex is involved in vesicle trafficking, particularly the tethering and spatial targeting of post-Golgi vesicles to the plasma membrane prior to vesicle fusion (Zhang et al., 2010). Exo70B2 is targeted by ubiquitin-mediated regulation, contributes to the attenuation of PAMP-induced signaling and is required for the immune response against various phytopathogens (Stegmann et al., 2012, 2013). Given that many MAPK substrates are destabilized upon phosphorylation (see above), the discovery of Exo70B2 as a putative phosphoprotein downstream of MPK3/MPK6 activation suggests Exo70B2 and possibly other exocyst proteins are likely to be direct substrates.

Taken together with another cluster of phosphoproteins associated with ER/Golgi secretion and protein glycosylation (Figure 2), exocyst/vesicle-mediated transport components appear to be “targeted” by MAPKs and thus coordinate vesicle-mediated translocation/secretion of their transported cargoes to the appropriate cellular compartment (i.e., key concept 2) or “site-of-action” for triggering defense reactions. Note that since the cargoes in these vesicles may not necessarily be only proteins, one may envisage that antimicrobial or signaling compounds may also be secreted by exocytosis and therefore contribute to chemical defense or signaling (see next key concept below).

## MAPK activation triggers chemical defense responses

Using a non-targeted LC/MS-based metabolite profiling approach, the accumulation kinetics of 113 mostly semi-polar secondary metabolites were detected after MPK3/MPK6 activation, of which the most prominent substances were tryptophan (Trp)-derived metabolites such as indole-3-carboxylic acid derivatives or characteristic defense metabolites (e.g., camalexin and indole glucosinolates) (Lassowskat et al., 2014). The timing of the accumulation of *de-novo* synthesized defense metabolites coincides perfectly with the activation profile of MAPKs and the levels of these metabolites are reduced or partially reduced in *mpk6* or *mpk3* backgrounds, respectively. This indicates a MAPK-mediated production of Trp-derived defense metabolites. Indeed, the fungal-responsive MAPK cascade ending in MPK3 and MPK6 has been shown to regulate camalexin production through transcriptional regulation of the corresponding biosynthetic genes in Arabidopsis (Ren et al., 2008). During fungal infection, the camalexin biosynthesis is regulated by MPK3/MPK6-mediated phosphorylation of WRKY33 (Mao et al., 2011), which targets the promoters of the biosynthetic genes directly to activate transcription (Birkenbihl et al., 2012). Note that our study (Lassowskat et al., 2014) confirmed phosphorylation of WRKY33 and its interacting protein partner, as well as other WRKYs after MPK3/MPK6 activation (see Key Concept 1 above).

Four clusters (marked in green, Figure 2) highlight putative phosphoprotein networks that could deliver precursors or catalyze the reactions for biosynthesis of the observed defense metabolites. These are: (1) Trp biosynthesis, (2) sulfur metabolism, (3) indolics/camalexin biosynthesis, and (4) proteins from the *PENETRATION* (*PEN*) genetic pathway required for biosynthesis and secretion of toxic defense molecules. Since, the gene expression for several of these proteins (e.g., *PAD3*) is induced by WRKY33 (Birkenbihl et al., 2012) or active MAPKs (Mao et al., 2011), their detection in the phosphoproteomics approach may reflect increased expression instead of (in)direct phosphorylation by MAPKs. However, at least for PEN2 and PEN4, there were no apparent changes (during the time course of the study) in protein levels prior to phosphoprotein enrichment but enhanced detection after phosphoprotein enrichment (Lassowskat et al., 2014). This may be taken as indirect evidence for the phosphorylation of PEN2 and PEN4 after MAPK activation. In particular, PEN4 is known to be phosphorylated and a T49A mutation near its catalytic site reduces its enzymatic activity (Wang et al., 2009). However, T49 of PEN4 and also most of the known phosphosites of PEN1 and PEN3 are not typical MAPK targeted sites (Nühse et al., 2003; Stein et al., 2006; Benschop et al., 2007). Nevertheless, PEN3 phosphorylation has been demonstrated in other proteomics studies (Stecker et al., 2014) and phosphorylation is important for its ABC transporter activity (Stein et al., 2006). It is thus possible that there are kinases activated downstream of the MAPKs (in our simulated MAPK activation system). Such MAPK-activated protein kinases (MAPKAP kinases) are known in animal p38 MAPK pathways (Ben-Levy et al., 1998) but have not been reported for plants. Irrespective of the regulation mode, our and other studies clearly showed that, even in the absence of any pathogen-derived signals, an artificial MAPK activation is sufficient to trigger defense metabolite production in Arabidopsis. Furthermore, in similar reports, constitutively-active MKKs were shown to lead to enhanced biosynthesis gene expression or production of phytoalexins in tobacco (Yang et al., 2001) and rice (Kishi-Kaboshi et al., 2010a,b), respectively. Thus, MAPKs are key *in vivo* regulators of chemical defense in plants **(Key Concept 3)**. The release of the toxic metabolites may either proceed through ABC transporters such as PEN3 or exocytosis through vesicles bearing defense compounds. In addition, the appearance of protein clusters for ATPases, acetyl-CoA biosynthesis or glycolysis (dark blue clusters, Figure 2) presumably serves to provide the energetic sources, proton gradient or metabolite co-factor activators of enzymes.

KEY CONCEPT 3. **MAPKs are key *in vivo* regulators of plant chemical defense in the cell (Green clusters in Figure 2).**

Even in the absence of pathogen signals, MAPK activation is sufficient to drive the production of antimicrobial substances that govern the outcome of plant resistance to pathogens.

## Other cellular signaling events are induced downstream of MAPKs and there is interplay of signaling pathways

Closely associated to the acetyl-CoA (fatty acid precursor) biosynthesis protein group is a cluster of proteins for phospholipid signaling (light blue cluster, Figure 2). These include phospholipase-C2 (PLC, AT3G08510), phospholipase-D-α (PLD-α, AT3G15730), PLD-γ (AT4G11850), and diacylglycerol kinase 5 (DGK5, AT2G20900) and are responsible for producing second messenger molecules such as diacylglycerol (DAG), inositol 1,4,5-trisphosphate (IP_3_) or phosphatidic acid (PA) (Munnik and Testerink, 2009; Ruelland et al., 2015). The fifth protein in this cluster, PTEN2a (AT3G19420) is a dual phosphatase with activity on both proteins and phosphoinositides. In *in vitro* assays, it actively dephosphorylates the 3′ phosphate group of PI_3_P (phosphatidylinositol 3-phosphate), PI_3, 4_P2 (phosphatidylinositol 3,4-bisphosphate), PI_3, 5_P2 (phosphatidylinositol 3,5-bisphosphate) but only poorly PI_3, 4, 5_P3 (phosphatidylinositol 3,4,5-trisphosphate), and furthermore binds PA with high affinity. In line with its stress-inducible expression, PTEN2s are proposed to be effectors of lipid signaling in plants (Pribat et al., 2012). A co-involvement of lipid signaling and MAPKs during stress has been reported previously (Munnik and Meijer, 2001). Thus, it seems that further cellular signaling pathways are activated downstream of the MAPKs **(Key Concept 4)**.

KEY CONCEPT 4. **Secondary downstream signaling events may be activated by MAPKs and there is interplay with other signaling pathways (light blue clusters in Figure 2).**

MAPKs apparently trigger phosphorylation of proteins (or accumulation of phosphoproteins) involved in phospholipid, ROS, redox, and phytohormone signaling. These contribute to cellular signal feedback loops and interplay between signaling pathways.

The lipid second messenger, PA, can activate MAPKs, which may act as a feedback amplification loop in signaling (Lee et al., 2001). PA binds and activates the PDK1 kinase that then phosphorylates the downstream oxidative stress-response protein kinase OXI1 (Anthony et al., 2006). Interestingly, OXI1 is required for full activation of MPK3/MPK6 after treatment with H_2_O_2_ (Rentel et al., 2004). While OXI1 acts as an upstream regulator of MPK3/MPK6 activation, MPK3/MPK6 can phosphorylate OXI1 *in vitro*. These findings suggest interplay between phospholipid signaling, reactive oxygen species (ROS) and MAPKs in complex feedback loops.

PLC inhibitor studies in tomato pinpoint a link between PA production and ROS accumulation upon MAMP treatment (Raho et al., 2011). MAPK cascades are also involved in regulating the ROS burst in response to pathogen attack in tobacco (Asai and Yoshioka, 2008). Similarly, in Arabidopsis, ROS homeostasis is mediated by a MAPK cascade consisting of MEKK1 and MPK4 (Nakagami et al., 2006). The cell death induced by constitutively-active MKKs is associated with ROS (Ren et al., 2002). These studies show a link between MAPK and ROS signaling. However, MPK3/MPK6 activation and NADPH oxidase-mediated ROS burst are two independent signaling events in plant immunity (Kroj et al., 2003; Xu et al., 2014). Much remains to be explored for the complex crosstalk between MAPK and ROS signaling. A recent proteome analysis of the *anp2 anp3* (MAPKKKs) double mutant showed a change in antioxidant response (Takac et al., 2014). In our study, a cluster of proteins involved in redox homeostasis (superoxide dismutase, peroxiredoxin-2D, 2-cysteine peroxiredoxin, and catalase 3) can also be detected (Figure 2). These may act to attenuate the MAPK-mediated ROS toxic effects or are part of MAPK-ROS signaling interplay.

The constitutively-active MKK system used in our study is known to induce ethylene biosynthesis (Kim et al., 2003; Liu and Zhang, 2004). While genetic evidence points to MAPK elements downstream of ethylene, the activation of MAPK cascades by ethylene is still intensely debated (Ecker, 2004; Hahn and Harter, 2009). Nevertheless, there is definitely signal interplay between ethylene biosynthesis, MAPK, ROS, calcium and calcium-dependent protein kinase (CDPK) signaling (Ludwig et al., 2005; Dubiella et al., 2013; Seybold et al., 2014). Besides ethylene, MAPK cascades are known to be involved in signaling of other defense regulating phytohormones such as auxin (Kovtun et al., 1998), jasmonates (Takahashi et al., 2007), and salicylic acid (Takahashi et al., 2007). Altogether, ethylene biosynthesis and the appearance of putative phosphoproteins with function in phospholipid signaling and redox regulation after MPK3/MPK6 activation can be taken as support for Key Concept 4 that additional signaling processes are triggered downstream of MAPK activation.

## Perspectives

As compared to typically transient MAPK activation by MAMPs (Asai et al., 2002; Bethke et al., 2012), our study (Lassowskat et al., 2014) is based on an artificial sustained MAPK activation system. One may question if such a system reflects *bona fide* immune MAPK signaling. The duration of MAPK activation is known to be a critical determinant for modulation of robustness of the immune signaling network and our system may perhaps reflect such prolonged MAPK activation seen during effector-triggered immunity (Tsuda et al., 2013). Nevertheless, the purpose of our study is to identify MPK3/MPK6 substrates and through the continuous presence of active MAPKs, may potentially identify substrates that are typically transiently phosphorylated or are unstable. We identified 538 putative MPK3/MPK6 substrates while a similar study identified 141 candidates (Hoehenwarter et al., 2012). Taken together with other protein array-based kinase screens for MAPK substrates (Feilner et al., 2005; Popescu et al., 2009), a plethora of putative MAPK substrates are available. Obviously such studies have their limitations and will not provide mechanistic insights into MAPK signaling prior to additional experimental investigations (Takáč and Šamaj, 2015). For instance, phosphorylation of some plant MAPK substrates can increase their enzymatic activities (Park et al., 2011) or transcription activation properties (Ishihama et al., 2011). Future work should first distinguish the “indirect” phosphoproteins from the direct MAPK substrates (e.g., as evaluated by direct *in vitro* kinase assays). Next, the role of phosphorylation on the function of individual substrate proteins can be used to dissect MAPK-mediated cellular signaling control.

Here, rather than emphasizing the impact of phosphorylation on individual substrates, we summarize the global implication (derived from our study) of plant MAPK activation in four key concepts. We propose that MAPKs orchestrate a (chemical) defense response through complex interplay with multiple signaling pathways, regulation of gene/protein expression (including protein stability control), and/or cellular component re-compartmentalization. At the same time, many of these protein networks highlighted here can be considered as “general” pathways (e.g., the translation/transcription components in Key Concept 1, re-compartmentalization events in Key Concept 2 or also the interplay with other signaling pathways in Key Concept 4), which are involved in many other signaling processes. Hence, a future challenge will be to understand how MAPKs coordinate between these general pathways toward specific signaling outcomes. For instance, as illustrated in our work, the specific regulation of plant chemical defense (Key Concept 3) after MAPK activation suggests a concerted action between the specific regulome for antimicrobial substance production and the general cellular events like transcription and translation. Hence, how is signal specificity managed by MAPKs besides regulating global cellular events? As MPK3 and MPK6 used in this study are also activated by developmental and abiotic signals, how is signal specificity maintained without erroneous crosstalk? These are some questions that the research efforts of the MAPK community should target—hopefully as well-coordinated as the MAPKs apparently do.

## Funding

Our research is supported by the German Research Foundation through the SFB 648 (TP-B1) project “Molecular mechanisms of information processing in plants” and the ERA-PG project “PathoNET” (SCHE 235/15-1). LE was supported through the BMBF project ProNET-T3 (03ISO2211B).

### Conflict of interest statement

The authors declare that the research was conducted in the absence of any commercial or financial relationships that could be construed as a potential conflict of interest.
